# Zoonotic origins and animal hosts of coronaviruses causing human disease pandemics: A review

**DOI:** 10.4102/ojvr.v87i1.1895

**Published:** 2020-12-21

**Authors:** Abdalla A. Latif, Samson Mukaratirwa

**Affiliations:** 1 School of Life Sciences, College of Agriculture, Engineering and Science, University of KwaZulu-Natal, Westville Campus, Durban, South Africa; 2 One Health Centre for Zoonoses and Tropical Veterinary Medicine, School of Veterinary Medicine, Ross University, St Kitts, Basseterre, West Indies

**Keywords:** coronaviruses, humans, Zoonosis, animal hosts, one health

## Abstract

The first known severe disease caused by a coronavirus (CoV) in humans emerged with the severe acute respiratory syndrome (SARS) epidemic in China, which killed 774 people during its 2002/2003 outbreak. The Middle East respiratory syndrome (MERS) was the second human fatal disease, which started in 2012 in Saudi Arabia and resulted in 858 fatalities. In December 2019, a new virus, SARS-CoV-2 (COVID-19), originating from China, began generating headlines worldwide because of the unprecedented speed of its transmission; 5.2 million people were infected and 338 480 had been reported dead from December 2019 to May 2020. These human coronaviruses are believed to have an animal origin and had reached humans through species jump. Coronaviruses are well known for their high frequency of recombination and high mutation rates, allowing them to adapt to new hosts and ecological niches. This review summarises existing information on what is currently known on the role of wild and domesticated animals and discussions on whether they are the natural reservoir/amplifiers hosts or incidental hosts of CoVs. Results of experimental infection and transmission using different wild, domesticated and pet animals are also reviewed. The need for a One Health approach in implementing measures and practices is highlighted to improve human health and reduce the emergence of pandemics from these zoonotic viruses.

## Introduction

Coronaviruses (CoVs) are a group of large single-stranded ribonucleic acid (RNA) viruses, belong to the order Nidovirales, family Coronaviridae, and are classified based on the difference in protein sequences into four genera: alpha, beta, gamma and delta CoVs (Woo et al. 2012). The morphology represents crown-like spikes on the outer surface of the virus seen under electron microscope, thus acquiring the name coronavirus. The Evolution model suggests that bat CoV serves as gene sources of alpha- and beta-CoVs, whilst wild bird CoV serves as gene sources of gamma and delta CoVs (Wang & Zhang 2016; Woo et al. 2012). The beta-CoVs include severe acute respiratory syndrome coronavirus (SARS-CoV), the Middle East respiratory syndrome coronavirus (MERS-CoV) and severe acute respiratory syndrome coronavirus-2 (SARS-CoV-2) (Chan et al. 2013). Coronavirsues of each genus are found in diverse animal species, including horses, cattle, pigs, dogs, cats, birds, rabbits, rats and ferrets and other wildlife animals, and some are infective in humans (Chan et al. 2013). They cause mild to severe diseases in animals and humans. Clinical signs and symptoms include respiratory, enteric, hepatic, renal and neurological manifestations, amongst other forms of the disease. It remains unclear as to how CoVs of each species have evolved, but different evolution models have been proposed, including a model where the bat CoVs serve as a gene source of all CoVs (Cui, Fang & Zheng-Li 2019). Coronavirsues have constantly crossed species barriers and some have emerged as important human pathogens (Cui et al. 2019).

Coronavirus species that cause important diseases in animals include avian infectious bronchitis virus, transmissible gastroenteritis virus in pigs, porcine epidemic diarrhoea virus and swine acute diarrhoea syndrome CoV. The first known severe disease caused by a CoV in humans emerged with the aevere acute respiratory syndrome (SARS) epidemic in China (Guan et al. 2003; Kuiken et al. 2003; Zhou et al. 2020), which killed 774 people during its 2002/2003 outbreak. The Middle East respiratory syndrome (MERS) was the second human disease, which started in 2012 in Saudi Arabia, resulting in 858 fatalities (Van Boheemen et al. 2012; Zaki et al. 2012). In December 2019, a new virus began to spread worldwide because of the high rate of its transmission; 5.2 million got infected and 338 480 died from December 2019 to May 2020 (WHO 2020). The disease is caused by an infection with a novel coronavirus SARS-CoV-2 and was named COVID-19, which stands for CoV disease 2019 (Guan et al. 2003; Zhou et al. 2020).

Coronaviruses have high mutation rates which allow them to adapt to new hosts and ecological niche (Cui et al. 2019). This review article discusses the animal origin and hosts of human coronaviruses (HCoVs), summarising existing information on what is currently known on the role of wildlife animals, domesticated and pet animals, and whether they are the natural reservoir hosts, intermediate hosts or incidental hosts of CoVs. It is important to determine the host range of the HCoVs for prevention and control purposes, risk assessments, development of diagnostic techniques, vaccines and therapeutics. The interdisciplinarity nature of a One Health approach presents an ideal opportunity to deal with such complex pandemics engaging professionals from different disciplines, including medical professionals, veterinary professionals, environmental health and social sciences (Kelly et al. 2017).

## Methodology

The search focus of this review was on the zoonotic origin and susceptibility of pet and wildlife animals to HCoVs. Literature was accessed from Google Scholar and MEDLINE/PubMed and the inclusion criteria in our literature search covered the period from the first occurrence of HCoV pandemic in 2002 to the current COVID-19 pandemic till September 2020, and included the following:

- The role of wild, domesticated and pet animals in the maintenance and transmission of the CoVs (search terms: ‘coronavirus transmission’, ‘wildlife’, ‘companion animals’, ‘livestock’)
- Results from experimental infection and transmission using different wild, domesticated and pet animals (search terms: ‘coronavirus transmission’, ‘experimental infection’ and ‘laboratory animals’).
- The potential of pet and wildlife animals acquiring CoV infection from infected persons who have had close contact with such animals (search terms: ‘coronavirus natural transmission’ and ‘human–pet animals/wildlife transmission’).

For this review, a reservoir host is defined as one in which the infectious agent normally lives and multiplies and is, therefore, a common source of infection to other animals; thus, it is frequently an ancestral/natural host. It harbours HCoV permanently and the virus is usually well adapted and non-pathogenic to the host. Because the infectious agent frequently depends upon the natural host for its long-term existence, the host is also called a maintenance host (Thrusfield 2005). An intermediate host is a new host, which becomes infected with HCoV from the natural/reservoir host before or at the time of its introduction to humans. The virus may be amplified in the intermediate host and in this case serves as the zoonotic source of human infection (Thrusfield 2005). An amplifier host is one in which the virus multiplies rapidly to high levels, providing an important source of infection. Such an animal, because of temporal increase in its population size, may suddenly increase the magnitude of the infecting virus (Thrusfield 2005). Incidental (dead-end or accidental) host is defined as a host that does not usually transmit the virus to other animals (Thrusfield 2005).

The aspects of HCoVs transmission, pathogenesis, genomic and vaccines studies, control and socioeconomics were excluded.

### Ethical consideration

This article followed all ethical standards for a research without direct contact with human or animal subjects.

## Results and discussion

### Severe acute respiratory syndrome

Search results showed reports which revealed that early infected human cases of SARS in 2002 and 2003 had history of contact with live game animal markets or handling meat for consumption in the Guangdong province of southern China (Zhong et al. 2003). The epidemiological linkage of the initial human cases to wild game animals suggested that SARS-CoV is zoonotic in origin (Guan et al. 2003).

#### Natural reservoir host

Following wide surveillance using next-generation sequencing performed in wild animals, over 200 novel CoVs have been identified in bats, and 35% of the bat virome sequenced is composed of CoVs (Banerjee et al. 2019; Woo et al. 2010). Generally, CoVs do not cause apparently overt disease in bats and this was confirmed following experimental infection. They have been identified as natural reservoir hosts for a number of highly lethal viral diseases in human and domestic animals (Fan et al. 2019). Bats are one of the oldest mammalian groups with a unique immune system, which allows them to be asymptomatic carriers of these viruses (Allocati et al. 2016; O’Shea et al. 2014). In addition, they have a long lifespan and can fly across large geographical regions. Bats are known as major evolutionary reservoirs and ecological drivers of CoVs diversity that may spill over to humans and cause disease (Banerjee et al. 2019; Martina et al. 2003; Tu et al. 2004). The contact rate amongst bats and between bats and humans or domestic animals is a major contributing factor to zoonotic virus transmission.

The novel CoVs related to human SARS-CoV (SARS-rCoVs) were discovered in horseshoe bats (genus *Rhinolophus*) in China and Hong Kong (Lau et al. 2005; Martina et al. 2003; Tu et al. 2004). These SARS-rCoVs showed a genome sequence identity of 88% – 90% amongst the group and 87% – 92% identity to human or palm civet SARS-CoV isolates (Ge et al. 2013; Li et al. 2005).

#### Intermediate hosts

Investigations were carried out on seven wild and one domestic animal species in a live animal market in southern China that supplied meat in restaurants in Guangdong province (Guan et al. 2003). Of the apparently healthy masked palm civets (*Paguma larvata*), which came from several different owners, four out of six tested positive for SARS-like virus. One raccoon dog (*Nyctereutes procyonoides*) was found to have the virus in faeces. Sera from the palm civets and the raccoon dog had neutralising antibody to the animal CoV (Tu et al. 2004). Similarly, sera from people who recovered from SARS inhibited the growth of virus from the civets (Lau et al. 2005). This cross-reaction is a strong indication that these viruses are similar and that these animals serve as intermediate hosts. Moreover, the full-length genome sequence of the animal CoV (S CoV) had 99.8% homology to the human SARS-CoV, indicating that they are closely related (Guan et al. 2003; Lau et al. 2010; Skowronski et al. 2005). Masked palm civets that had not been exposed to live animal markets tested negative for SARS-CoV, implying that palm civets serve as the intermediate amplifying host but not the natural reservoir of SARSCoV (Cheng et al. 2007; Song et al. 2005; Ye et al. 2020).

#### Experimental animal infection

Palm civets (*Paguma larvata*) were shown to be susceptible and exhibited symptoms after experimental infection by SARS-CoV (Guan et al. 2003). Chinese ferret badgers (*Melogale moschata*), raccoon dogs and domestic cats (*Felis domesticus*) were also susceptible to infection by SARS-CoV (Martina et al. 2003). Cats remained asymptomatic, although some of the infected ferrets died of the disease. A primate model using cynomolgus macaques (*Macaca fascicularis*), which showed clinical and pathological features with some similarities to those found in humans, proved the theory (Koch’s postulate) that SARS-CoV is the causative agent of SARS (Cheng et al. 2007; Fouchier et al. 2003; Martina et al. 2003). Golden Syrian hamsters (*Mesocricetus auratus*) were found suitable for immune-prophylaxis and treatment studies as clinical signs were accompanied by high levels of viral titres and histopathological lesions (Roberts et al. 2005). Similarly, inoculated common marmosets generally had mild clinical disease and histopathological changes of pneumonia with extrapulmonary dissemination and high levels of viral replication in affected tissues (Greenough et al. 2005). Pigs and chickens are not susceptible to SARS-CoV (Weingartl et al. 2004). An important observation of these experimental animal infections is the diverse range of mammalian species that are susceptible to infection by SARS-CoV, which again demonstrated that SARS-CoV is highly capable of jumping interspecies barriers and is an excellent candidate as an emerging or re-emerging zoonotic pathogen (Cui et al. 2019).

These findings suggest that the wild animal markets provided a venue for the animal SARS CoV-like virus to amplify and transmit to new hosts, including humans. A mass cull of civet cats was undertaken for disease control in China in early 2004 following re-emergence of human cases with a possible link to these animals (Skowronski et al. 2005). It is not, however, clear whether any single animal species or different species of these animals is the intermediate host in the wild (Cheng et al. 2007; Vijayanand, Wilkins & Woodhead 2004). These results also throw light on the animal origin and evolution of SARS-CoV at the beginning of the SARS epidemic.

### Middle East respiratory syndrome

The MERS caused by a novel CoV (MERS-CoV) was first isolated in the Kingdom of Saudi Arabia in 2012 (Cotton et al. 2014; Zaki et al. 2012).

#### Natural reservoir host

Middle East respiratory syndrome-related CoVs have been found in several bat families, including Vespertilionidae, Molossidae, Nyteridae and Emballonuridae from the Middle East, Africa, the Americas, Asia and Europe (Anthony et al. 2013; Fan et al. 2019; Ithete et al. 2013; Memish et al. 2013; Mohd, Al-Tawfiq & Memish 2016). A CoV from Egyptian tomb bats, *Taphozous perforates*, showed 100% nucleotide identity to virus from the human index case patient (Memish et al. 2013). Ithete et al. (2013) reported the identification of a South African bat-derived CoV from *Neoromicia zuluensis* bat that has the closet phylogenetic relationship with MERS-CoV, termed PML/2011. This PML/2011 differed from MERS-CoV by only one amino acid exchange and is the closest to MERS-CoV. A CoV with 96.5% amino acid identity to MERS-CoV was found in a *Nyctinomops laticaudatus* bat from Mexico (Anthony et al. 2013). *Nycteris gambiensis*, from Ghana, was found to carry 2c beta-CoVs (Mohd et al. 2016). The same authors screened *Pipistrellus* bat species from Europe, where 14.7% were found to carry the 2c beta-CoV. Both 2c beta-CoVs isolated are genetically very closely related to MERS-CoV (Mohd et al. 2016). All types of bat MERS-related CoVs have been identified in the Vespertilionidae bat family and in multiple species in China (Fan et al. 2019). The MERS coronavirus infection in humans related to human–camel contact has only occurred in Saudi Arabia and not in any other continent. Overall, this relatedness of the CoV found in different bat species indicates that MERS-CoV originated from bats (Annan et al. 2013; Cui et al. 2019).

#### Intermediate host

Dromedary camels have been found to harbour the same MERS-CoV and MERS-CoV-like antibodies as humans and to shed the virus in high numbers in secretions from the upper respiratory tract (Adney et al. 2014). Cross-reactive antibodies to MERS-CoV have been found in dromedary camels in Oman, Canary Islands and Egypt (Perera et al. 2013; Reusken et al. 2013). In addition, a fatal case of human MERS-CoV infection was transmitted through contact with an infected camel with rhinorrhoea, and the full genome sequence of the isolates from the patient and the camel was identical (Azhar et al. 2014). It has been reported that a high proportion of dromedaries at a slaughterhouse shed nasal MERS-CoV, with a high risk of human exposure and potential of driving the epidemic (Farag et al. 2015).

The detection of MERS-CoV-specific antibodies with high prevalence in camels from the Middle East, Africa and Asia, the high genetic stability of the virus in camel populations and the high genetic identity between human and camel strains point to the evidence that MERS-CoV is perfectly adapted to camels and has been spread freely in this domesticated animal for a long time (Muller et al. 2014). Camels experimentally infected with MERS-CoV showed only mild symptoms but with massive virus shedding through respiratory route and faecal-oral route. Middle East respiratory syndrome coronavirus adapts in camels and maintains long-term endemicity and, therefore, the intermediate host can be defined as a natural secondary reservoir host with a spillover human infection (Ye et al. 2020). Approximately 55% of primary MERS-CoV cases are caused as a result of direct contact with dromedary camels or camel products (Conzade et al. 2018).

#### Experimental animal infection

Several animal species have been experimentally infected with MERS-CoV, including rhesus macaques, cynomolgus macaques, marmosets, ferrets, mice, Syrian hamsters, rabbits and dromedary camels (Adney et al. 2014; Falzarano et al. 2014; Yao et al. 2014). The outcome and development of lower respiratory tract disease varied in these animal models. Rhesus macaques were infected with MERS-CoV using intratracheal inoculation. The infected monkeys showed clinical signs of the disease (mild to moderate respiratory disease) with virus replication in lungs, lymph nodes, upper respiratory tract, histological lesions, and neutralising antibody production, indicating that this monkey model is suitable for studies of MERS-CoV infection (Yao et al. 2014). The infection of marmosets revealed broncho-interstitial pneumonia and viral antigen detected in the lungs and had viral titres a 1000-fold higher than that in rhesus macaques (Falzarano et al. 2014; Kuiken et al. 2003; Skariyachan et al. 2019). The clinical symptoms in the common marmoset model were mild to severe respiratory disease (Falzarano et al. 2014; Skariyachan et al. 2019). New Zealand white rabbits have been proved to shed MERS-CoV from their upper respiratory tract, but remained asymptomatic after MERS-CoV inoculation (Skariyachan et al. 2019; Zaki et al. 2012). Infected transgenic mice showed severe respiratory disease, weight loss and 100% mortality (Agrawal et al. 2015). Jamaican fruit bats infected with MERS-CoV had the virus detected in their respiratory and intestinal tracts for 9 days, with no overt signs of the disease (Munster et al. 2016).

There is no evidence of sustained human-to-human transmission in the community and no evidence of airborne transmission as main routes of transmission from all information available from recent MERS-CoV cases (WHO 2019b). In epidemiological terms, MERS-CoV was not considered of pandemic potential (Table 1). Cases resulting from animal-to-human transmission will continue to occur and will eventually lead to limited community transmission within households. Infected camels shed MERS-CoV through nasal and eye discharge, faeces, milk, meat and urine. Such camels may not show any signs of infection, which places a great risk in any contacts made with camels that can potentially infect humans. Consistent application of adequate prevention and control measures has been used to prevent virus transmission (WHO 2019b).

**TABLE 1:** Summary of epidemiological data for severe acute respiratory syndrome coronavirus, Middle East respiratory syndrome coronavirus and severe acute respiratory syndrome coronavirus 2.

Epidemiological/biological characteristics	SARS-CoV	MERS-CoV	SARS-CoV-2
Transmissibility, date of outbreak	Pandemic, 2002/2003	Epidemic† 2012	Pandemic, 2019
Origin of outbreak	Guangdong province, China	Saudi Arabia	Wuhan, Hubei province, China
Natural reservoir host	Horseshoe bats	Horseshoe bats	Horseshoe bats
Intermediate host	Probably palm civet, raccoon dogs, Chinese ferret badger‡	Camel/dromedary	Probably pangolins
Acquired/incidental host	Chinese ferret badgers, raccoon dog	Not known‡	Zoo tiger, lion, mink, cats, dog
Experimental positive transmission	Palm civets, Chinese ferret badgers, domestic cats, cynomolgus macaques, Golden Syrian hamsters, common marmosets	Rhesus macaques, common marmoset, New Zealand white rabbits, Transgenic mice	Ferrets, cats, dogs
Human transmission	Human-to-human	Camel/Human-to-human	Human-to-human
Total positive global infections	8096	2499	5 229 444
Total global death	774	858	338 480
Case fatality rate	9.6%	34.5%¶	6.5%§

MERS-CoV, Middle East respiratory syndrome coronavirus; SARS-CoV, severe acute respiratory syndrome coronavirus; SARS-CoV-2, severe acute respiratory syndrome coronavirus-2.

† , MERS-CoV was not considered of pandemic potential.

‡ , No MERS-CoV pathogenesis was observed in small animals.

¶ , Cheng et al. (2007), WHO (2019a).

§ , WHO (2020).

### Severe acute respiratory syndrome coronavirus 2

The ongoing novel coronavirus outbreak is the third highly infectious disease emerging at the animal–human interface, causing considerable concern and disruption as it spreads across the world (Murdoch & French 2020). The index cases were epidemiologically linked to seafood and live wholesale animal market in Wuhan, Hubei province, China, in November 2019. The world case fatality rate for COVID-19 is 6.9%, higher than that of the influenza H1N1 2009 pandemic (< 1%) and is low compared to 10% for SARS-CoV and 34.5% for MERS-CoV (Table 1). The WHO (2020) declared the outbreak as a public health emergency of international concern (pandemic) on 30 January 2020.

#### Natural reservoir host

Genomic analysis revealed that SARS-CoV-2 is phylogenetically related to severe acute respiratory syndrome-like (SARS-like) bat viruses; therefore, bats could be the possible primary reservoir (Hu et al. 2017; Zhou et al. 2020). Severe acute respiratory syndrome-coronavirus 2 belongs to the species SARS-rCoV together with SARS-CoV from humans and SARS-rCoVs from horseshoe bats (Gorbalenya et al. 2020; Wu et al. 2020), concluding that SARS-CoV-2 has a bat origin. According to genome sequences available so far, the virus most closely related (96.2% of nucleotide sequence identity) to SARS-CoV-2 is strain BatCoVRaTG13 identified from a bat, *Rhinolophus affinis*, from Yunnan province, China, followed by SARS-rCoVs identified from pangolins (Tang et al. 2020).

#### Intermediate host

The pangolin (*Manis javanica*) was found to harbour a CoV, beta-CoVs, which is strikingly homologous to SARS-CoV-2. This indicates that pangolins might serve as one of the intermediate hosts or that the pangolin beta-CoVs could contribute gene fragments to the final version of SARS-CoV-2 (Contini et al. 2020). Scientists have been working to identify the source of SARS-CoV-2 (Cyranoski 2020; Lam et al. 2020; Lau et al. 2020; Xiao et al. 2020), and high-throughput sequencing of pangolins tissues obtained before the occurrence of COVID-19 pandemic revealed the presence of CoV sequences that fall into the SARS-CoV-2 lineage (Lam et al. 2020). The genome sequence of one virus isolate had very high similarity (99.83% – 99.92%) to genomic organisations to SARS-CoV-2. Another study suggested that SARS-CoV-2 might be a recombinant virus, with its genome backbone evolved from Yunnan bat-virus-like SARSr-CoVs and from the pangolin-virus-like SARSr-CoVs (Cyranoski 2020). The discovery of multiple lineages of pangolin CoV and their similarity with SARSCoV-2 suggest that pangolins should be considered as possible intermediate hosts in the emergence of novel CoVs and should be removed from wet markets to prevent zoonotic transmission.

#### Incidental hosts

**Cats:** Previous reports have shown that SARS-CoV can infect cats (Martina et al. 2003), implying that they might also be susceptible to SARS-CoV-2. Chinese researchers investigated the serological prevalence of SARS-CoV-2 in pet cats after the outbreak by an indirect enzyme-linked immunosorbent assay (ELISA) and virus neutralisation test (Zhang et al. 2020). The results indicated that 14.7% (15/102) of the cat blood samples collected after the outbreak tested positive for the presence of antibodies specific for SARS-CoV-2. Three cats owned by COVID-19-affected patients had the highest levels of antibodies and this might suggest a cat–human transmission of SARS-CoV-2 and further studies are urgently needed to confirm this.

Human–pet cat transmission has been reported from several countries. A pet cat in Spain whose owner had recently died of COVID-19 tested positive for SARS-CoV-2 by reverse transcription-polymerase chain reaction (RT-PCR) (American Veterinary Medical Association 2020; Segalésa et al. 2020). In France, SARS-CoV-2 RNA was detected in samples collected from a cat whose owner was suspected to have COVID-19. A second SARS-CoV-2-positive cat had developed clinical signs of respiratory disease, including cough, and was examined several times. The nasopharyngeal sample was positive for SARS-CoV-2 (American Veterinary Medical Association 2020; Fritz et al. 2021). Two pet cats with confirmed cases of SARS-CoV-2 infection in New York had clinical signs of a mild respiratory illness. The owner of the first cat assumed to be an asymptomatic carrier and the second cat owner had tested positive for COVID-19 before the cat became ill (American Veterinary Medical Association 2020; Newman et al. 2020). In Belgium, SARS-CoV-2 RNA was detected by RT-PCR and high-throughput sequencing PCR in the faeces and vomit of a cat with digestive and respiratory clinical signs. The cat was owned by a person confirmed to be infected with SARS-CoV-2 (American Veterinary Medical Association 2020; Brown 2020). Using RT-PCR, a cat in Germany was confirmed to be infected with SARS-CoV-2. The owner had died of COVID-19 (American Veterinary Medical Association 2020).

**Zoo tigers and lions:** The United States Department of Agriculture’s (USDA) National Veterinary Services Laboratories confirmed that a 4-year-old Malayan tiger at New York’s Bronx Zoo tested SARS-CoV-2 positive (USDA 2020). This is the first report of a tiger being infected with COVID-19. Samples from the tiger were taken and tested after several lions and tigers at the zoo showed symptoms of respiratory illness and decreased appetite. However, only samples from the tiger were taken for diagnosis.

**Dogs:** Dogs belonging to COVID-19-infected owners were screened for SARS-CoV-2 during the outbreak in China (Sit et al. 2020). Two out of 15 dogs from households with confirmed human cases of COVID-19 were found to be infected using quantitative RT–PCR, serology, sequencing the viral genome, and in one dog, virus isolation. Severe acute respiratory syndrome-coronavirus 2 RNA was detected from nasal and oral swabs. Both dogs had seroconverted using neutralisation assays. Viral genetic sequences of viruses from the two dogs were identical to the virus detected in the respective human cases. The animals remained asymptomatic during quarantine. The results suggest that there are instances of human-to-animal transmission of SARS-CoV-2, although it is unclear whether infected dogs can transmit the virus to other animals or to humans.

**Minks:** Four mink farms have been reported positive for SARS-CoV-2 in the Netherlands since the start of the outbreak (Oreshkova et al. 2020; American Veterinary Medical Association 2020), and there are concerns about the potential for widespread transmission of the virus. The affected farms had reported an increased incidence of gastrointestinal and respiratory disease, including fatal infections, with pregnant minks developing more serious disease with increased mortality. The disease was confirmed in sick minks by SARS-CoV-2-specific PCR assay. It was believed that the virus was transmitted to minks by COVID-19-infected farm caretakers; however, mink-to-mink transmission could have spread the virus within each farm.

### Experimental animal infection

**Ferrets:** The Chinese ferret badgers (*Melogale moschata*) was infected with SARS-CoV and showed clinical signs and efficient transmission between infected and naïve controls (Roberts et al. 2005). Based on these results, ferrets were considered as suitable animal models for SARS-CoV-2, and hence studies to use ferrets as an experimental animal model to evaluate treatments and vaccines for the disease in humans were performed by Kim et al. (2020). The authors have shown in a comprehensive study that ferrets can be infected experimentally with SARS-CoV-2, can shed virus in body discharges and transmit the infection to other naïve ferrets. High levels of viral RNA were detected in nasal washes of all infected ferrets, between 2 and 8 days after COVID-19 inoculation. Three naive ferrets were placed in contact with the infected ones, and they all became infected, showing that the virus is transmissible between ferrets. Ferrets appear to be susceptible to infection but less so to disease and no mortality. Infectious virus was detected from the nasal washes of all ferrets but not from the rectal swabs. Antibodies against SARS-CoV-2 were detected in all ferrets by an ELISA and a neutralisation assay test. Ferrets have already been used as models in influenza studies (Bouvier 2015), and several laboratories have started COVID-19 research using these highly susceptible animals.

**Cats:** In an infectivity study carried out under laboratory conditions (Shi et al. 2020), viral RNA was detected in the upper and lower respiratory tracts and in the small intestine of two cats that were euthanised on days 3 and 6 post-infection. Infectious virus was detected in the respiratory system, and droplet transmission had occurred between cats. All virus-inoculated cats and one exposed cat had developed antibody tested by ELISA and neutralisation assays. In SARS-CoV-2 direct contact transmission study (Halfmann et al. 2020), three domestic cats were inoculated with the virus and each was co-housed with a naïve cat 1 day after inoculation of the virus. Nasal and rectal swab specimens were taken daily and tested for infectious virus on Vero-E6 cells. The virus was detectable in all three inoculated cats and the three co-housed cats. All the cats remained asymptomatic and all had high IgG antibody titres.

**Dogs:** In a SARS-CoV-2 infectivity and direct transmission study under controlled conditions (Shi et al. 2020), five 3-month-old dogs were inoculated intra-nasally and co-housed with two uninoculated dogs. Pharyngeal and rectal swab specimens were collected from days 2 to 14 post-infection. Viral RNA was positive in the rectal swabs; however, infectious virus and viral RNA were not detected in any swabs, organs or tissues collected from a euthanised positive dog. Only two dogs out of four of the inoculated dogs had positive antibody titres, whilst the two contact dogs tested seronegative. These results concluded that dogs are less susceptible to SARS-CoV-2.

**Other domestic and wildlife animals:** Susceptibility of pigs, chickens and ducks to SARS-CoV-2 was investigated by using the virus intra-nasal inoculation methodology as that used to assess cats and dogs (Shi et al. 2020). Infected animals were housed in the same room with their uninfected species to investigate the transmission of the virus. Viral RNA was not detected in any swabs collected from the inoculated animals or from naïve contact animals. Viral RNA was not detected in all organs examined in euthanised pigs and all the animals were seronegative. These results indicate that pigs, chickens and ducks are not susceptible to SARS-CoV-2.

**One Health approach to control CoV disease 2019 pandemic:** To prevent the spread of the novel COVID-19, the Chinese government banned the trade in wild animals. A similar measure was also introduced in 2003 for SARS, but this control measure was not sustained. Continuous surveillance of mammals is essential for a better understanding of the ecology of CoVs and for the prevention of animal-to-human transmission of the disease (Zhou et al. 2020).

With reports of positive transmission of SARS-CoV-2 from humans to domestic cats and to tigers and lions at the Bronx Zoo (USDA 2020), coupled with these experimental data showing the ease of transmission between domestic cats, there is a public health need to recognise and further investigate the potential chain of human–cat–human transmission. This is of importance, given the potential for SARSCoV-2 transmission between family members in households with cats whilst living under ‘lockdown’ regulations. Moreover, cats may be passive intermediate host of SARS-CoV-2 because infected cats may not show any appreciable symptoms that might be recognised by their owners. Given the need to stop the COVID19 pandemic through various mechanisms, including breaking transmission chains, a better understanding of the role cats and other domesticated animals may play in the transmission of SARS-CoV-2 to humans is needed and emphasises the importance of closer collaboration amongst various scientific disciplines and stakeholders following the One Health approach.

## Conclusion

Three pandemics caused by the highly pathogenic HCoV of zoonotic origin occurred during 2002, 2012 and 2019. Table 1 summarises the epidemiological data for SARS-CoV, MERS-CoV and SARS-CoV-2. In the case of MERS-CoV, there is no evidence of sustained human-to-human transmission in the community and no evidence of airborne transmission as main routes of transmission (WHO 2019a). In contrast, the diversity of animal species susceptible to SARS-CoV and SARS-Cov-2 strongly suggests a jump of these viruses to cross species barrier. Based on current information, the three HCoVs that are presumed to have originated from their natural reservoir hosts, the bats and their intermediate hosts have also been identified. The intermediate hosts for SARS-CoV proven to date are the masked palm civets and raccoon dogs. This pandemic was controlled by the elimination of the intermediate host, palm civets, from live markets in China. Middle East respiratory syndrome coronavirus closely related to bat CoV were identified in countries in four continents, which implied that the natural reservoir host originated from bats. However, none of these geographical areas investigated, except for Saudi Arabia, is known for MERS-CoV primary infection in humans. The high prevalence of antibody titres, the high genetic stability of the virus and the high genetic identity between camel and human strain are all evidence that MERS-CoV is perfectly adapted and maintained amongst camels over the years. Therefore, camels are intermediate hosts in this situation and can be referred to as natural secondary reservoir hosts. A phylogenetic analysis showed that SARS-CoV-19 significantly clustered with a sequence from the bat SARS-like CoV isolated in 2015 and that the transmission chain began from the bat (Benvenuto et al. 2020). Severe acute respiratory syndrome-coronavirus 2-related CoVs were identified in Malayan pangolins, which suggests that pangolins should be considered as possible intermediate hosts in the emergence of SARS-CoV-19. Incidental infections, experimental infectivity and transmission findings from laboratory studies have shown that, of the animal species screened and investigated so far, cats are the most susceptible species for COVID-19. Public Health and Veterinary Services should work together using a One Health approach to share information and conduct a risk assessment when a person with COVID-19 reports being in contact with companion animals or other animals. It is recommended that RT-PCR should be used to test oral, nasal and faecal/rectal samples of such animals. However, there is no conclusive evidence that domestic and wildlife animals can actively transmit SARS-CoV-2 to humans. On the other hand, scientific research and epidemiological investigations are yet to start.
